# Preparing Effective InterProfessional Teams to Meet the Needs of Older Adults in Integrated Healthcare Settings

**DOI:** 10.1093/geroni/igab046.3270

**Published:** 2021-12-17

**Authors:** Karen Bullock, Kim Stansbury, David Fitzpatrick, Gloria Anderson

**Affiliations:** 1 North Carolina State University, Raleigh, North Carolina, United States; 2 NCSU, RALEIGH, North Carolina, United States

## Abstract

Background: Gerontology education has evolved from focusing primarily on the individual practitioner outcomes to promoting integrated, inter-professional team approaches to integrated care. Practicum training and service learning are effective pedagogy for paraprofessionals in integrated care settings to support clinicians and advance their effectiveness in meeting the needs of older adults. Interprofessional education (IPE) aims to enhance the capacity of practitioners to work collaboratively as integrated team members. Yet, little is known about the implementation of IPE in colleges and universities that are not affiliated with a medical center or medical school. This presentation will describe the implementation of IPE in a School of Social Work without a medical school system. We will explore students’ experiences with the implementation, facilitation, and evaluation of this workforce development model, and will discuss both challenges and successes. Methods: Narrative data collected over the past two-year period with social work student participants in IPE reflect their perspectives on the selection process for the specialized training, placement in an interprofessional integrated care setting, and the learning experience. Participants represented cohorts of more than 200 trainees. Results: Challenges of effective interprofessional health care teams include the approach taken to integrating the teams, level of knowledge and skills required to be an effective team member, and the need for supervision within integrated care experiences. Successes include increased professional self-efficacy. Participants reported post-completion of IPE, (1) desirable outcomes achieved for their patients, such as care satisfaction and role satisfaction for themselves as practitioners.

